# Investigation of Electropolishing for High-Gradient 1.3 GHz and 3.9 GHz Niobium Cavities

**DOI:** 10.3390/ma17133207

**Published:** 2024-07-01

**Authors:** Yue Zong, Jinfang Chen, Dong Wang, Runzhi Xia, Jiani Wu, Zheng Wang, Shuai Xing, Xiaowei Wu, Xuhao He, Xiaohu Wang

**Affiliations:** 1 Shanghai Institute of Applied Physics, Chinese Academy of Sciences, Shanghai 201800, China; zongyue@sinap.ac.cn (Y.Z.); wangdong@sinap.ac.cn (D.W.); wangzheng@sinap.ac.cn (Z.W.); 2 University of Chinese Academy of Sciences, Beijing 100049, China; 3 Shanghai Advanced Research Institute, Chinese Academy of Sciences, Shanghai 201210, China; wujn@sari.ac.cn (J.W.); xings@sari.ac.cn (S.X.); 4 School of Physical Science and Technology, ShanghaiTech University, Shanghai 201210, China; xiarzh2023@shanghaitech.edu.cn (R.X.); hexh2022@shanghaitech.edu.cn (X.H.); wangxh4@shanghaitech.edu.cn (X.W.); 5 Zhangjiang Laboratory, Shanghai 201210, China; wuxw@zjlab.ac.cn

**Keywords:** electropolishing, cold EP, SRF cavity

## Abstract

Electropolishing (EP) has become a standard procedure for treating the inner surfaces of superconducting radio-frequency (SRF) cavities composed of pure niobium. In this study, a new EP facility was employed for the surface treatment of both 1.3 GHz and 3.9 GHz single-cell cavities at the Wuxi Platform. The stable “cold EP” mode was successfully implemented on this newly designed EP facility. By integrating the cold EP process with a two-step baking approach, a maximum accelerating gradient exceeding 40 MV/m was achieved in 1.3 GHz single-cell cavities. Additionally, an update to this EP facility involved the design of a special cathode system for small-aperture structures, facilitating the cold EP process for 3.9 GHz single-cell cavities. Ultimately, a maximum accelerating gradient exceeding 25 MV/m was attained in the 3.9 GHz single-cell cavities after undergoing the cold EP treatment. The design and commissioning of the EP device, as well as the electropolishing and vertical test results of the single-cell cavities, will be detailed herein. These methods and experiences are also transferable to multi-cell cavities and elliptical cavities of other frequencies.

## 1. Introduction

Superconducting materials, which are characterized by their low surface resistance and hysteresis loss, enable SRF cavities to operate with minimal energy consumption and withstand higher power operation, thus demonstrating attractive features in continuous-wave (cw) particle accelerators. Currently, SRF cavities are primarily fabricated from high-purity niobium, and the surface conditions directly affect their radio-frequency performance.

Following the fabrication of bare cavities, the inner surfaces usually exhibit insufficient smoothness and even defects, thereby constraining the RF performance of cavities. To optimize RF performance, the inner surface of SRF cavities should closely approximate the theoretical ideal. Generally, the initial 100–200 µm of material can be removed via processes such as buffered chemical polishing (BCP) or electropolishing (EP) [1]. The EP on a bulk Nb cavity is characterized by the elimination of microroughness (leveling) and the absence of crystallographic and grain boundary attack (brightening), resulting in the production of smooth, bright surfaces [2,3,4,5]. Thus, in contrast to BCP, EP offers a smoother surface and potentially improved RF performance [6,7,8,9,10,11]. The EP process was first introduced for cavity surface treatment by Diepers [12]. The EP technique with rotating niobium cavities was pioneered by researchers at KEK [13]. Afterward, the EP process was successfully applied to polish SRF cavities worldwide by several institutes, such as JLab [14], FNAL [15,16,17], ANL [18], DESY [10], and IHEP [19]. In 2017, a novel approach termed “cold EP” was introduced by researchers at Fermilab, demonstrating the ability to achieve a smoother surface and uniform material removal throughout the cell [16,17]. Particularly for cavities treated with a nitrogen-doping recipe, the attainment of a smoother surface is of paramount importance [20,21,22].

The SHINE project requires 600 1.3 GHz nine-cell TESLA cavities and 16 3.9 GHz nine-cell cavities with exceptional performance [23]. For the 1.3 GHz nine-cell cavities, the target is to achieve an intrinsic quality factor ($Q_{0}$) of $2.7\times 10^{10}$ at 16 MV/m, along with a maximum accelerating gradient ($E_{acc}$) exceeding 19 MV/m. Similarly, for the 3.9 GHz nine-cell cavities, the objective is to attain a $Q_{0}$ value of $2.0\times 10^{9}$ at 13.1 MV/m, with the maximum $E_{acc}$ surpassing 16.5 MV/m. Hence, the implementation of the cold EP process is imperative in realizing high accelerating gradients, especially for 1.3 GHz nine-cell cavities. In 2019, we undertook an upgrade of a simple EP device located in Ningxia, China, incorporating an outer surface cooling system. This device was subsequently employed in nitrogen-doping experiments conducted on single-cell cavities [24,25]. Starting in 2021, a novel platform for the surface treatment of SRF cavities known as the Wuxi platform was constructed in Wuxi, China. The newly built EP devices at the Wuxi platform have been used in high-Q recipe studies, including those of nitrogen doping and medium temperature baking [26,27]. This paper primarily presents the newly established EP apparatus at the Wuxi platform, which was employed for the surface treatment of 1.3 GHz and 3.9 GHz single-cell cavities. Utilizing the cold EP mode and a two-step baking process [28,29,30,31], we achieved an elevated accelerating gradient for 1.3 GHz single-cell cavities.

## 2. EP devices at SHINE

### 2.1. EP Device for a Single-Cell Cavity

**Figure 1:**
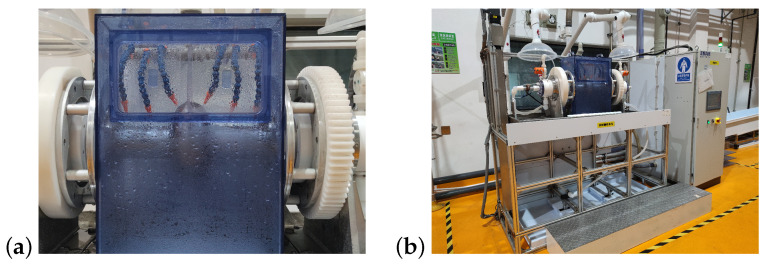
(**a**,**b**) EP apparatus for 1.3 GHz single-cell cavities at the Wuxi Platform.

In 2021, two novel EP devices were constructed for the SHINE project on the Wuxi platform. One is the single-cell EP device (referred to as a “small EP”), as depicted in Figure 1; it possesses the capacity to accommodate the treatment processes for 1.3 GHz single-cell cavities and for 3.9 GHz single-cell and nine-cell cavities. The other one is specifically tailored for the treatment of 1.3 GHz nine-cell cavities [26]. Both EP devices share the same acid-mixing system.

**Figure 2:**
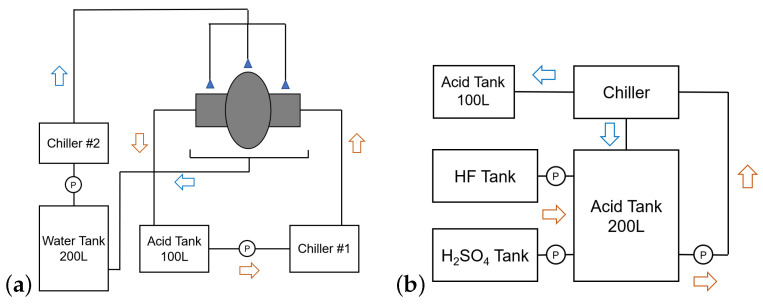
Schematics of single-cell EP device; (**a**) the polishing system and (**b**) the acid-mixing system.

One of the most important objectives of the new EP system is to achieve steady temperature control. This is achieved by utilizing separate chiller units to independently regulate the temperatures of the external cooling water and the acid. The schematic of the single-cell EP device at the Wuxi platform is depicted in Figure 2a. Chiller 1 controls the acid temperature, while Chiller 2 regulates the cavity’s external surface temperature. During the cold EP mode, the temperature of the cavity’s external wall can be maintained below 8 °C, and the acid outlet temperature at both ends of the cavity can be maintained around 10 °C. Figure 1b illustrates the spray piping for external surface cooling water.

The acid mixture ratio is based on the established classical proportions. The ratio of the electrolyte is $H_{2}{\mathrm{SO}}_{4}$(98%):HF(48%) = 1:9 by volume. However, the newly designed EP system could automatically mix acid based on volume ratios, and a schematic diagram of the acid-mixing system is shown in Figure 2b. During the acid-mixing process, a separate refrigeration unit is employed for acid temperature control, thereby mitigating HF evaporation. The mixed acid is pumped into acid tanks in preparation for the EP treatment. To polish a 1.3 GHz single-cell cavity, around 60 L of electrolyte is needed every time. When the niobium concentration of the electrolyte is about 10 g/L, the used acid is dumped, and fresh acid is pumped into the acid tank. For light EP, fresh acid is used every time.

**Figure 3:**
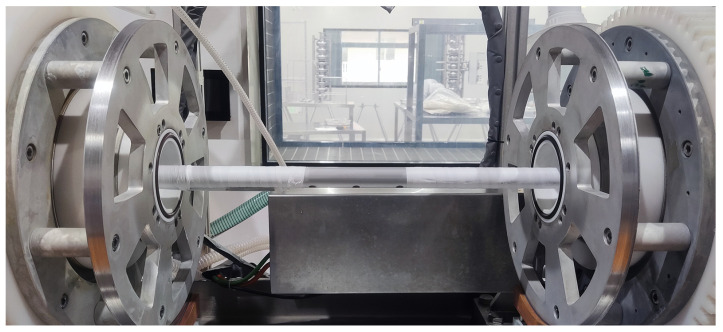
Cathode rod for the EP process of a 1.3 GHz single-cell cavity.

Both ends of the cathode rod are enveloped in polytetrafluoroethylene (PTFE) tape, exposing only the central region where the acid outlet is located, as shown in Figure 3. The diameter of this cathode rod is 24 mm. The area ratio between the cathode and anode is about 1:5 without PTFE tape and 1:10 during the EP process with PTFE tape. Overflow baffles are also installed at both ends of the device, and the cathode rod passes through the middle of the overflow baffle. When the acid level inside the cavity exceeds the height of the overflow baffle, the acid overflows and enters the EP return pipe, thereby maintaining the acid level inside the cavity at around 60%.

### 2.2. Parameters of the EP Device

**Figure 4:**
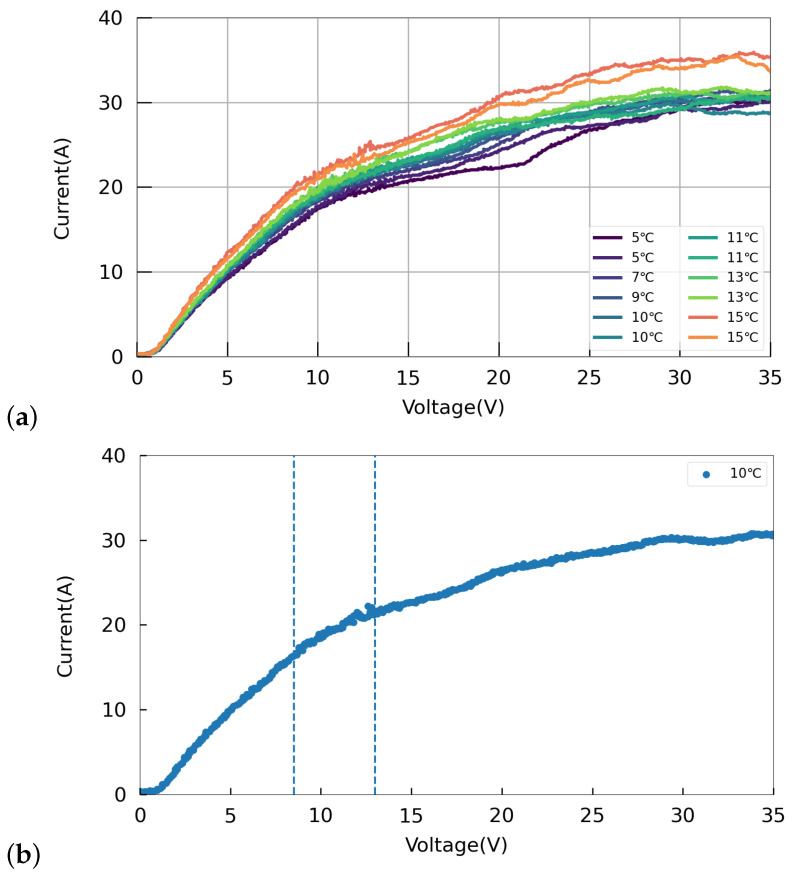
I–V curves of the EP device for a 1.3 GHz single-cell cavity. (**a**) I–V curves across acid temperatures ranging from 5 to 15 °C. (**b**) I–V curve at an acid temperature of 10 °C.

In order to find the proper working point to polish a 1.3 GHz single-cell cavity, we measured the current–voltage (I–V) curves at different acid temperatures. Figure 4 shows the I–V curves for acid temperature ranging from 5 to 15 °C. To monitor the inlet and outlet acid temperatures, three acid temperature probes were strategically placed at the center of the cathode and at both ends of the cavity beam pipes. As shown in Figure 4a, with an increase in acid temperature, the reaction became more vigorous, leading to a slight uptick in the current. It can also be observed that there was no distinct plateau region in the curve, indicating an increase in the current even within the polishing region, which was consistent with the observations reported by Bertucci et al. [32]. Figure 4b, which presents the I–V curve at an acid temperature of 10 °C, shows that the voltage range from 0 to 8.5 V corresponded to a clear etching region, while the range from 8.5 to 13 V represented an oscillation region. Based on these observations, an operating voltage of 15 V was selected to ensure optimal polishing performance.

Previous studies [17] indicated that a proper temperature for cold EP polishing should be below 12 °C. Therefore, we chose 10 °C as the controlled temperature during the polishing process. The temperature control system adjusted the temperature of the inlet acid based on the feedback from the temperature probe inside the cavity, thereby maintaining the acid temperature inside the cavity at around 10 °C during the polishing process. Our experimental results indicated that an acid flow rate of 3 L/min was sufficient to control the acid temperature around 10 °C throughout the EP process. The parameters employed for the EP of 1.3 GHz single-cell cavities are detailed in Table 1 below.

**Figure 5:**
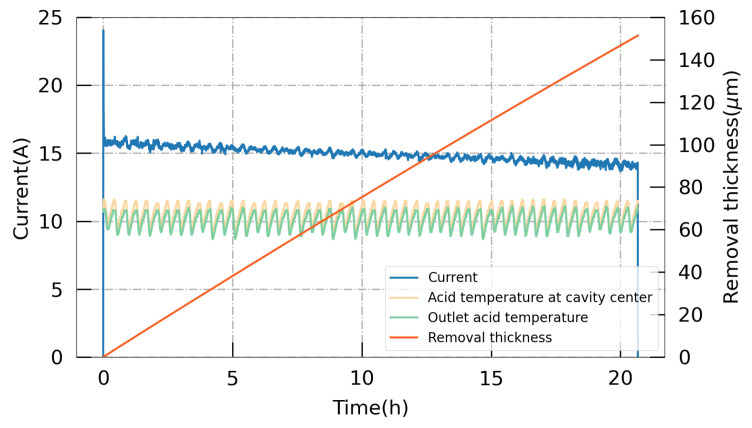
Recorded data on the current and temperature during the bulk EP process, along with the cumulative polishing amount calculated in real time.

**Figure 6:**
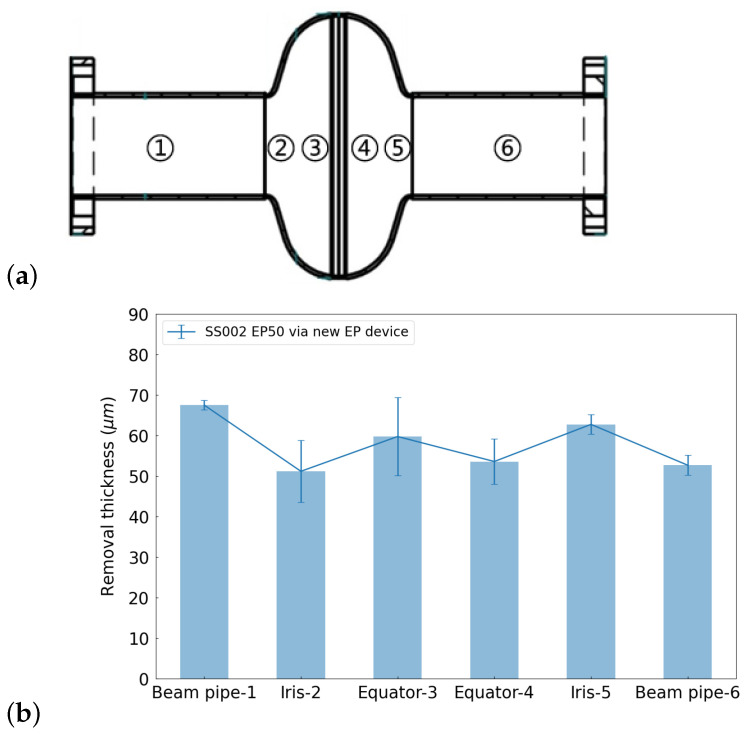
(**a**) Scheme of a 1.3 GHz single-cell cavity with the locations of the thickness measurement points. Points 1 and 6 correspond to measurement points on the beampipe, points 2 and 5 near the iris area, and points 3 and 4 on the equator area. (**b**) Measurement of the removal thickness with an ultrasonic probe at various positions of a 1.3 GHz single-cell cavity before and after 50 um polishing; the removal data at the six positions depicted in the figure represent the averages of measurements taken at four points of axial symmetry.

During the EP process, the data on the current, temperature, and acid flow rate can be monitored and recorded in real time. With Equation (1), the theoretical removal thickness can be calculated with the current, where *I* is the current, $S_{0}$ is the inner surface area, *M* is the molecular weight of niobium at *M* = 92.9 g/mol, ρ is the density of niobium at ρ=8.57 g/cm^3^, and *F* is the Faraday constant. Thus, the average removal rate during the cold EP process for a 1.3 GHz single-cell cavity is around 7 μm/h or 0.12 μm/min. The typical curves for 150 μm bulk EP are summarized in Figure 5. From an examination of the complete curves, it is evident that the polishing process was stable and controllable. Furthermore, the final polished thickness aligned closely with the measurements obtained from the ultrasonic thickness gauge. Figure 6 shows a thickness removal measurement of targeted 50 μm along a 1.3 GHz single-cell cavity with an ultrasonic thickness gauge, demonstrating a removal non-uniformity of less than 20% at the cell part.

(1) $$d\left[\frac{\mu m}{s}\right]=\frac{I}{S_{0}}\cdot \frac{M}{5\rho F}$$

## 3. Surface Treatment of 1.3 GHz Single-Cell Cavities

### 3.1. The 1.3 GHz Single-Cell Cavity Treatment

**Figure 7:**
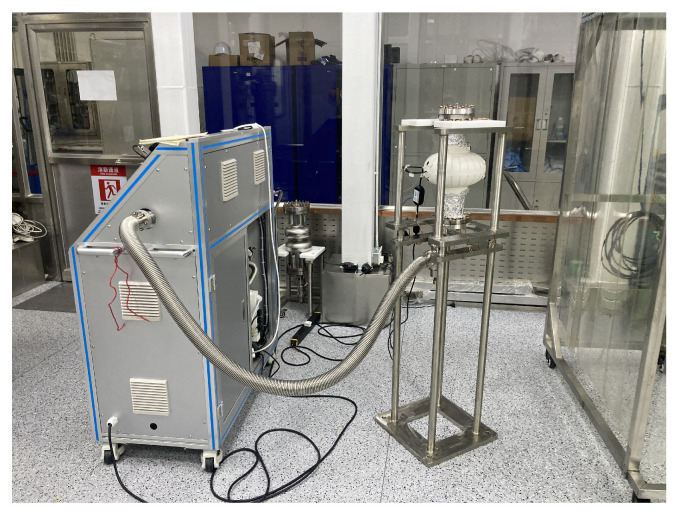
A 1.3 GHz single-cell cavity undergoing the two-step baking process.

**Figure 8:**
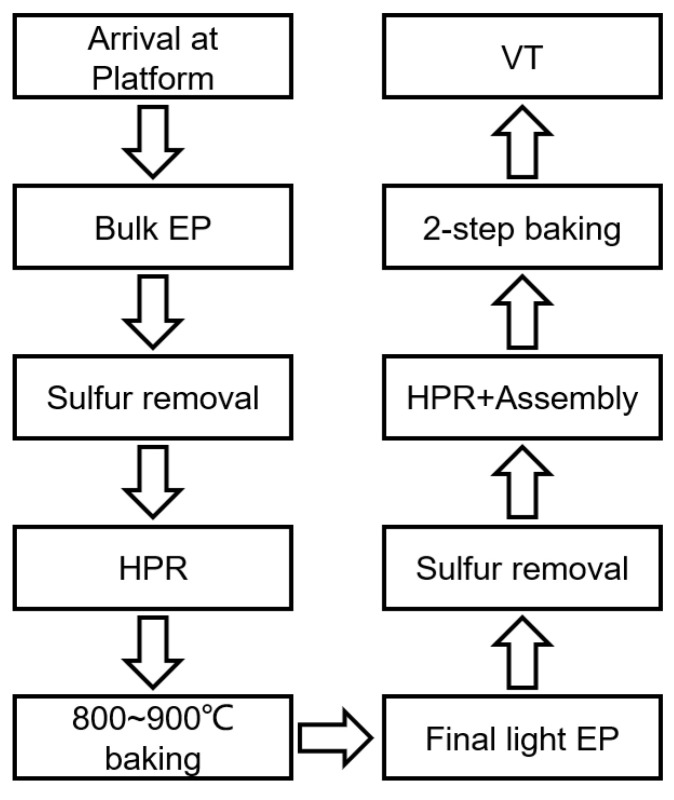
The main process flow for 1.3 GHz single-cell cavities.

Two 1.3 GHz single-cell cavities, namely, S02 and S04, were subjected to an EP baseline experiment. These two cavities were previously used for other studies. Initially, 50 μm removal was performed to reset the surfaces of both cavities. Subsequently, after undergoing a 900 °C baking process to release hydrogen and enhance the magnetic flux capabilities, a final 20 μm light EP process was applied to achieve the desired surface. Throughout the entire EP procedure, the cold EP mode was employed, and fresh acid was used for the final light EP process. These cavities were then baked in situ with a two-step baking method, consisting of baking at 75 °C for 4 h, followed by ramping up the temperature to 120 °C and baking for an additional 48 h. The equipment used in the two-step baking process is shown in Figure 7. The cavity was connected to the vacuum pump to maintain a high vacuum state during the baking process, and the heater enveloped the central region to bake the cavity. To further assess the recipe’s stability, two newly manufactured cavities, SS002 and SS003, underwent treatment with the same procedure, but the bulk EP removal thickness was 150 μm. The main processing flow is illustrated in Figure 8.

### 3.2. Optical Inspection of the Inner Surface

**Figure 9:**
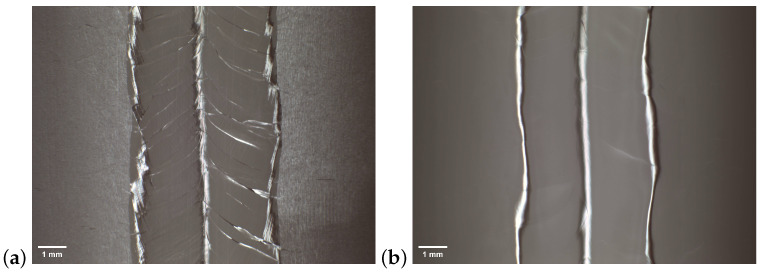
Inspection results of SS002 (**a**) before EP and (**b**) after 150 μm bulk EP.

After the bulk EP and high-temperature baking, the inner surface was examined with an inspection camera that is widely used in many SRF labs, such as KEK, DESY, and IHEP [19,33,34]. By comparing the inner surface before and after treatment, we were able to efficiently examine the quality of the EP process. Typically, the camera was set to take a picture for every four degrees, and it took 90 pictures in total for one circle. The pictures used for the inspection of SS002 are summarized in Figure 9 and show that the inner surface was mirror-like after bulk EP.

### 3.3. Vertical Test Results for the 1.3 GHz Single-Cell Cavities

Following the surface treatment, the cavities underwent a vertical test at 2 K to evaluate their RF performance, which was primarily assessed via Q-E curves. Typically, such testing is carried out using a low-temperature vertical test stand (VTS).

**Figure 10:**
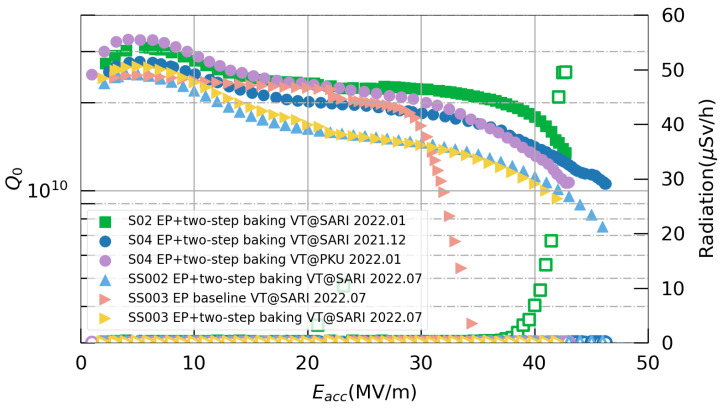
Vertical test results for the 1.3 GHz single-cell cavities at 2 K.

After clean assembly in a clean room, the cavities underwent the two-step baking process and a vertical test. The vertical test results for the 1.3 GHz single-cell cavities are shown in Figure 10. The cavity SS003 was tested once before the two-step baking process, and the maximum $E_{acc}$ value was 34.5 MV/m, with a strong high-field Q-slope that was limited by quenching. After the two-step baking process, the maximum $E_{acc}$ value of SS003 was increased to 42 MV/m. The maximum $E_{acc}$ values of all four cavities were over 40 MV/m and were limited by quenching; S04 and SS002 reached 46 MV/m, which verified the effectiveness of the treatment—especially the EP process. The Q-E curves of SS002 and SS003 exhibited differences from those of S02 and S04, with slightly lower $Q_{0}$ values being observed in the cavities of the SS series. A similar bifurcation of Q-E curves was also noted in a study by Bafia et al. [31], occurring within the same cavity during different tests. They primarily attributed this phenomenon to the suppression of niobium nano-hydride formation. However, in our instance, the niobium materials used in the cavities of the SS series and S series were from different suppliers, which could account for this discrepancy.

In order to verify the reliability of the vertical test results, the cavity S04 was sent to PKU for a comparison. This cavity was transferred to PKU directly after completing the vertical test at SARI while maintaining a vacuum inside. According to the results, S04 achieved similar results at both SARI and PKU, with a difference of less than 10%. It can be observed that there were still some differences between the two Q-E curves from PKU and SARI. SARI recorded a higher maximum $E_{acc}$ value of 46 MV/m, whereas the maximum $E_{acc}$ value recorded at PKU was slightly smaller at 43 MV/m. However, at PKU, higher $Q_{0}$ values at medium and low fields were observed. We mainly attribute this to the differences in the fast cooling processes employed by the two facilities during testing.

## 4. Surface Treatment of 3.9 GHz Single-Cell Cavities

### 4.1. Updates of the EP Device for 3.9 GHz Single-Cell Cavities

**Figure 11:**
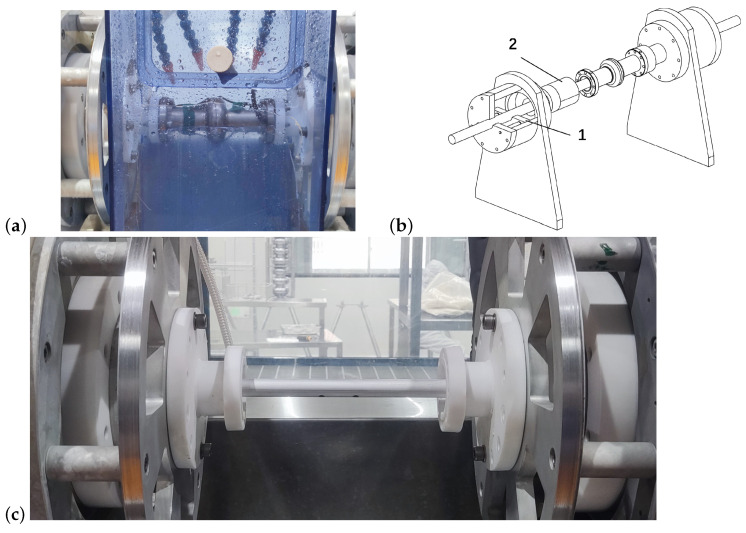
(**a**) Electropolishing of a 3.9 GHz single-cell cavity, (**b**) the schematic design of the cathode system to adapt to the electropolishing of the 3.9 GHz cavity, including adapters and overflow plates, and (**c**) the cathode for 3.9 GHz single-cell cavities.

Due to their smaller dimensions, modifications to the EP equipment were necessary to meet the requirements for electrochemical polishing of the 3.9 GHz superconducting cavities. To address this, two adapter pieces made of PVDF material were initially designed and fabricated. These adapters facilitated the connection of the 3.9 GHz single-cell cavity bundle with the EP equipment, and this was supplemented by the addition of a cable to maintain electrical connectivity between the superconducting cavity and the equipment, as shown in Figure 11a. Furthermore, the overflow plates at both ends of the equipment underwent redesigning. Considering the compatibility of the narrower cathode rod with the 3.9 GHz superconducting cavity, the new design aimed to enhance the stability during the insertion of the cathode rod while also ensuring overflow functionality, thereby meeting the requirements for controlling the acid level within the cavity during the EP process. Figure 11b shows a sketch of this new design, where position 1 represents the overflow baffle, and position 2 denotes the limiting block. Simultaneously, adjustments were made to the cathode rod, primarily reducing its diameter to 16 mm, and the number of acid outlet holes on the cathode rod was reduced to 2 because of the shorter cell size of the 3.9 GHz cavity. However, due to space constraints, PTFE tape was not wrapped around the cathode rod. The actual installed configuration can be observed in Figure 11c.

In the design of the cathode rod for the 3.9 GHz cavity, the initial objective was to maintain a comparable surface area ratio to that of the 1.3 GHz cavity by proportionally reducing the diameter of the cathode rod to approximately 10 mm. However, it was observed that a cathode rod with a diameter of 10 mm and an inner diameter of only 4 mm posed challenges in adjusting and stabilizing the acid flow rate. Consequently, the diameter of the cathode rod was increased to 16 mm while maintaining an inner diameter of approximately 10 mm, thereby allowing for a broader range of acid flow rate adjustments.

**Figure 12:**
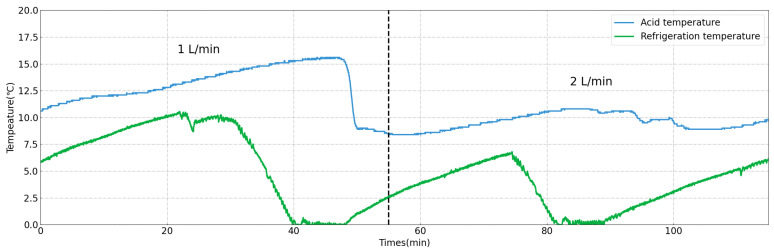
Temperature measurement curves across varying acid flow rates.

Furthermore, concerning the selection of the temperature and acid flow rate, efforts were made to uphold consistent polishing conditions akin to those of the 1.3 GHz cavity, namely, maintaining a temperature control of 10 °C and a proportional acid flow rate. However, experimentation revealed that different acid flow rates significantly affected temperature control. Figure 12 illustrates real-time temperature measurement curves across varying acid flow rates. The term ’acid temperature’ in this figure refers to the temperature measured with the temperature control probe, which was positioned at the outlet and continuously provided real-time feedback to the refrigeration unit, enabling adjustments to its cooling power. Conversely, the ’refrigeration temperature’ denotes the temperature of the refrigerant, as measured with an internal temperature probe within the refrigeration unit. This parameter reflects the operational temperature of the refrigeration unit at a given time and directly influenced the temperature of the acid as it entered the cavity. Notably, setting the temperature control to 10 °C resulted in a discernible delay in temperature regulation at a flow rate of 1 L/min, leading to an increase in the acid temperature to 15 °C before promptly decreasing. This fluctuation posed challenges for temperature control during the polishing process. Conversely, at a flow rate of 2 L/min, the temperature remained more stable around 10 °C.

The reasons for this phenomenon can be attributed to two main factors. On the one hand, a decrease in the acid flow rate resulted in more heat absorption per unit volume of acid, leading to an increase in the outlet acid temperature. On the other hand, cooler acid from the acid tank also required a longer time to reach the inner cavity, thereby prolonging the adjustment time of the temperature control system. Our experimental results indicate that an acid flow rate of 2 L/min is more suitable for the 3.9 GHz single-cell cavity, as it maintains a more stable acid temperature.

**Figure 13:**
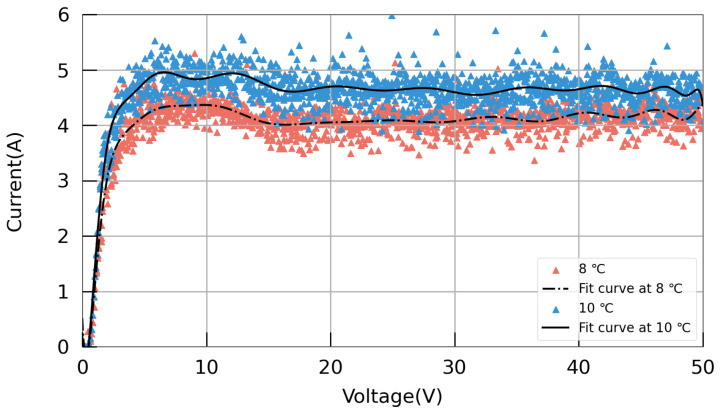
I–V curves of the EP device for the 3.9 GHz single-cell cavity.

The I–V curves of the 3.9 GHz cavity at acid temperatures of 8 and 10 °C are shown in Figure 13. The I–V curves exhibit a distinct plateau region, where the range from 0 to 4 V corresponds to the etching region, and that from 4 to 17 V represents the oscillation region. To avoid approaching the oscillation region too closely, a working voltage of 20 V was selected. The EP working parameters of 3.9 GHz single-cell cavities are shown in Table 1.

### 4.2. Optical Inspection of the Inner Surface

**Figure 14:**
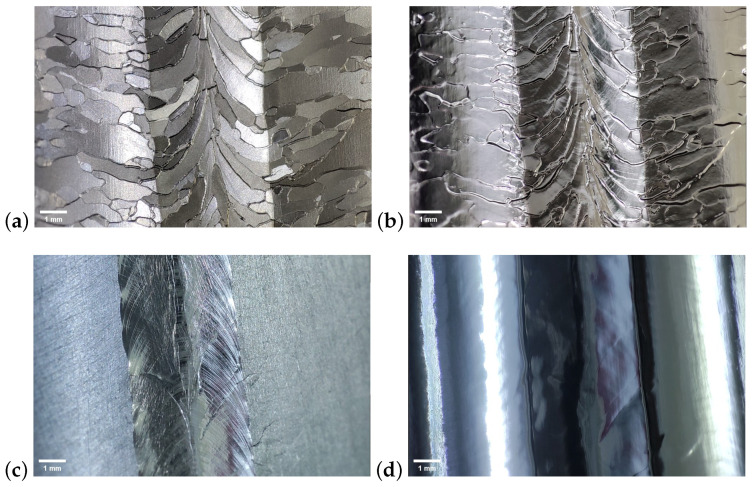
Optical inspection of 3.9 GHz single-cell cavities. (**a**,**b**) are from the fine-grain cavity H-S01, and they were shot before and after 150 um bulk EP, respectively; (**c**,**d**) are from the large-grain cavity H-L08, and they were shot before and after 150 um bulk EP, respectively.

To examine the internal surface conditions of the superconducting cavities before and after polishing, we devised a novel endoscopic optical inspection system tailored for the 3.9 GHz superconducting cavities. Utilizing a macro lens, the internal surfaces of the cavities could be clearly observed. Figure 14 illustrates comparative photographs of a 3.9 GHz cavity made of large-grain niobium before and after the EP process. It is evident from these images that, after polishing, the internal surfaces exhibited enhanced smoothness. Especially for large-grain cavities, the polished surfaces exhibited a noticeable mirror effect. It should be noted that fine-grain cavities appeared to have slightly poorer surface smoothness, which was most likely due to the previous BCP polishing treatment of these cavities, resulting in more prominent grain boundaries near the equator. After EP polishing, these grain boundaries still stood out, but the microroughness was significantly improved.

### 4.3. Vertical Test Results for the 3.9 GHz Single-Cell Cavities

**Figure 15:**
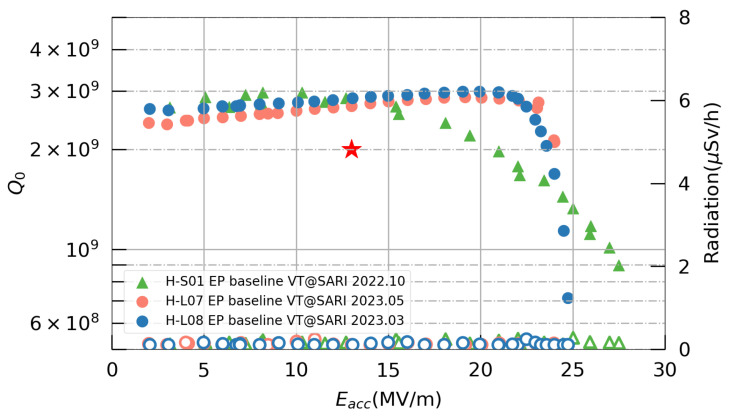
Vertical test results for the 3.9 GHz single-cell cavities.The red star represents the SHINE specification for the $Q_{0}$ value at 13 MV/m.

After determining the cold EP parameters for the 3.9 GHz single-cell cavity through experimentation, an EP baseline treatment was conducted on one fine-grained cavity, H-S01, and two large-grained cavities, H-L07 and H-L08. The procedure outlined in Figure 7 was followed, but the two-step baking process was omitted. Subsequently, vertical tests were conducted at SARI, and the results are summarized in Figure 15. All three cavities exhibited maximum accelerating gradients exceeding 23 MV/m, with the $Q_{0}$ values at 13 MV/m surpassing $2.7\times 10^{9}$, thus meeting the SHINE specifications of a $Q_{0}$ value greater than $2.0\times 10^{9}$ at 13 MV/m and a maximum accelerating gradient exceeding 16.5 MV/m. The Q-E curves of the two large-grained cavities showed a similar trend, which was characterized by a slight increase in $Q_{0}$ in the mid-field region, followed by a sudden decrease from 23 MV/m. Conversely, for the fine-grained cavity H-S01, $Q_{0}$ gradually declined starting from 15 MV/m. The underlying reasons for these differences warrant further investigation.

## 5. Discussion

We took notice of the significant influence of cathode rods on electrochemical polishing and cavity performance [35], which is particularly noteworthy for nine-cell cavities. In the current selection of EP parameters, we initially determined the diameter of the cathode rod and the position of the acid outlet on it. In the EP process for the 3.9 GHz single-cell cavity, the current polishing uniformity was not as good as that for the 1.3 GHz single-cell cavity. The main difference was that the absence of PTFE tape wrapped around the cathode rod in the EP process for the 3.9 GHz cavity contributed to a relatively large exposed area. Additionally, although the working voltage was chosen based on the I–V curves, it was comparatively higher than that in the 1.3 GHz EP process, exacerbating heat generation during the reaction. Drawing from our current experimental experience, the cold EP treatment of the 3.9 GHz cavity, given its smaller size, poses challenges in acid circulation and temperature control in comparison with the 1.3 GHz cavity. Therefore, in the future, we also hope to further optimize the working voltage and cathode rod size through sample experiments and simulations to enhance the polishing uniformity and cavity performance. Relevant experiments are currently in progress.

**Figure 16:**
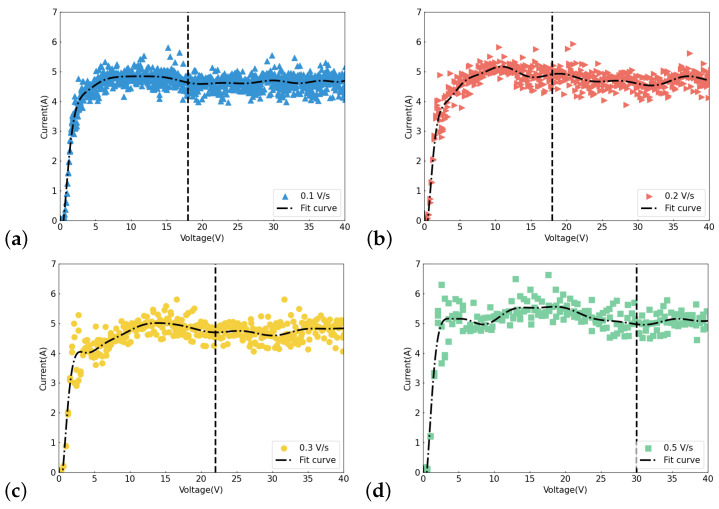
I–V curves with different voltage steps: (**a**) 0.1 V/s, (**b**) 0.2 V/s, (**c**) 0.3 V/s, (**d**) 0.5 V/s.

During the process of testing the I–V curves, we observed that, aside from the area ratio of the electrodes affecting the measured curve, the rate at which voltage was applied during testing could also cause a shift in the current plateau. As illustrated in Figure 16, applying a voltage at a rate of 0.1 V/s resulted in an onset voltage—at which the EP plateau appeared—of 17 V, while applying at 0.5 V/s led to an onset voltage of 25 V. We speculate that this may be due to the larger voltage step causing instability in the testing process; hence, a shorter voltage step is still required when measuring the I–V curve. Similar conclusions were also mentioned in Chouhan’s study [35], where they maintained 12 s at each voltage step to obtain more accurate I–V curves. In our testing procedure, due to the inability to adjust the interval time for each step, we gradually increased the voltage with smaller steps. On average, the voltage was increased by 1 V every 10 s.

## 6. Conclusions

A new small EP device was successfully installed and operationalized on the Wuxi platform. Based on its experimental outcomes, the system demonstrated its remarkable proficiency in controlling polishing process temperatures and ensuring the uniformity of inner cavity surface polishing. Additionally, the facility is capable of accommodating EP polishing for both 1.3 GHz single-cell cavities and 3.9 GHz single-cell cavities, making it more versatile in practical applications. Optical inspection of the inner surfaces showed obvious improvements in smoothness after EP. When utilizing this device for cold EP treatment followed by a two-step baking process, all four 1.3 GHz single-cell cavities achieved a maximum $E_{acc}$ that was higher than 42 MV/m, with half of them surpassing 46 MV/m. Furthermore, 3.9 GHz single-cell cavities subjected to cold EP treatment without the two-step baking process also exhibited elevated $E_{acc}$ values exceeding 25 MV/m.

## 7. Patents

CN202311431082 was published on 12 January 2024 and is currently undergoing substantive examination.

## Figures and Tables

**Table 1:** EP parameters chosen to polish 1.3 GHz and 3.9 GHz single-cell cavities.

Parameters	1.3 GHz Single-Cell Cavity	3.9 GHz Single-Cell Cavity
Voltage	15 V	20 V
Current	∼15 A	∼4 A
Electrolyte temp.	10 °C	10 °C
Average removal rate	∼6 μm/h	∼10 μm/h
Electrolyte flow rate	3 L/min	2 L/min
Cavity rotation speed	1 rpm	
Cooling water temp.	7 °C	

## Data Availability

The datasets generated and analyzed in the current study are available from the corresponding author upon reasonable request.

## Abbreviations

The following abbreviations are used in this manuscript:

EP	Electropolishing
SRF	Superconducting radio frequency
BCP	Buffered chemical polishing
